# Comparison of the SAWNUC model with CLOUD measurements of sulphuric acid‐water nucleation

**DOI:** 10.1002/2015JD023723

**Published:** 2016-10-27

**Authors:** Sebastian Ehrhart, Luisa Ickes, Joao Almeida, Antonio Amorim, Peter Barmet, Federico Bianchi, Josef Dommen, Eimear M. Dunne, Jonathan Duplissy, Alessandro Franchin, Juha Kangasluoma, Jasper Kirkby, Andreas Kürten, Agnieszka Kupc, Katrianne Lehtipalo, Tuomo Nieminen, Francesco Riccobono, Linda Rondo, Siegfried Schobesberger, Gerhard Steiner, António Tomé, Daniela Wimmer, Urs Baltensperger, Paul E. Wagner, Joachim Curtius

**Affiliations:** ^1^ Goethe University FrankfurtInstitute for Atmospheric and Environmental SciencesFrankfurt am MainGermany; ^2^ CERNGenevaSwitzerland; ^3^ Now at Institute for Atmospheric and Climate ScienceETH ZurichZürichSwitzerland; ^4^ SIMUniversity of Lisbon, University of Beira InteriorLisbonPortugal; ^5^ Paul Scherrer InstituteVilligenSwitzerland; ^6^ School of Earth and EnvironmentUniversity of LeedsLeedsUK; ^7^ Finnish Meteorological InstituteAtmospheric Research Centre of Eastern FinlandKuopioFinland; ^8^ Department of PhysicsUniversity of HelsinkiHelsinkiFinland; ^9^ Helsinki Institute of PhysicsUniversity of HelsinkiHelsinkiFinland; ^10^ Faculty of PhysicsUniversity of ViennaViennaAustria; ^11^ Now at Cooperative Institute for Research in Environmental SciencesUniversity of Colorado Boulder, Boulder, Colorado, USA and Chemical Sciences Division, NOAA Earth System Research LaboratoryBoulderColoradoUSA; ^12^ Now at Department of Applied PhysicsUniversity of Eastern FinlandKuopioFinland; ^13^ Now at Department of Atmospheric SciencesUniversity of WashingtonSeattleWashingtonUSA; ^14^ Institute of Ion Physics and Applied PhysicsLeopold‐Franzens UniversityInnsbruckAustria

**Keywords:** ion‐induced nucleation, binary nucleation

## Abstract

Binary nucleation of sulphuric acid‐water particles is expected to be an important process in the free troposphere at low temperatures. SAWNUC (Sulphuric Acid Water Nucleation) is a model of binary nucleation that is based on laboratory measurements of the binding energies of sulphuric acid and water in charged and neutral clusters. Predictions of SAWNUC are compared for the first time comprehensively with experimental binary nucleation data from the CLOUD chamber at European Organization for Nuclear Research. The experimental measurements span a temperature range of 208–292 K, sulphuric acid concentrations from 1·10^6^ to 1·10^9^ cm^−3^, and distinguish between ion‐induced and neutral nucleation. Good agreement, within a factor of 5, is found between the experimental and modeled formation rates for ion‐induced nucleation at 278 K and below and for neutral nucleation at 208 and 223 K. Differences at warm temperatures are attributed to ammonia contamination which was indicated by the presence of ammonia‐sulphuric acid clusters, detected by an Atmospheric Pressure Interface Time of Flight (APi‐TOF) mass spectrometer. APi‐TOF measurements of the sulphuric acid ion cluster distributions ( ${\left(H_{2}{\text{SO}}_{4}\right)}_{i}\cdot {\text{HSO}}_{4}^{-}$ with i = 0, 1, ..., 10) show qualitative agreement with the SAWNUC ion cluster distributions. Remaining differences between the measured and modeled distributions are most likely due to fragmentation in the APi‐TOF. The CLOUD results are in good agreement with previously measured cluster binding energies and show the SAWNUC model to be a good representation of ion‐induced and neutral binary nucleation of sulphuric acid‐water clusters in the middle and upper troposphere.

## Key Points

- Particle formation rates from SAWNUC model and CLOUD measurements at 208‐248 K generally agree
- Comparison of formation rates at 278‐298 K is limited due to ammonia contamination issues
- Modeled and measured cluster ion distributions agree, differences are likely due to fragmentation

## 1 Introduction

New particle formation is an important source of cloud condensation nuclei [*Kerminen et al.*, 2005; *Laaksonen et al.*, 2005; *Kuang et al.*, 2009]. The main compound responsible for new particle formation in the upper troposphere is considered to be sulphuric acid [*Weber et al.*, 1999]. Therefore, a large number of experimental studies of binary homogenous nucleation of sulphuric acid and water have been made, mostly limited to room temperature [see, for example, *Raes and Janssens*, 1986; *Wyslouzil et al.*, 1991; *Ball et al.*, 1999; *Young et al.*, 2008; *Zollner et al.*, 2012]. *Ball et al.* [1999] showed that small amounts of ternary vapor, in this case ammonia, can strongly enhance the observed nucleation rates. *Kirkby et al.* [2011] gave a more detailed quantification of this effect for temperatures between 248 and 292 K. In general, all laboratory experiments studying atmospheric aerosol nucleation are potentially prone to enhanced nucleation rates due to contaminants. Therefore, the concentrations of contaminants and their possible contribution to new particle formation rates need to be monitored carefully.

Besides ternary vapors, ions my also enhance the formation of molecular clusters in the atmosphere, as shown originally by *Wilson* [1898]. *Ney* [1959] therefore hypothesized that cosmic rays could affect clouds and thereby influence climate. Subsequent work has shown that ion‐induced aerosol nucleation is expected to account for a substantial fraction of new particle production in the free troposphere [*Raes et al.*, 1986; *Lovejoy et al.*, 2004; *Kirkby et al.*, 2011].

Modeling of nucleation processes is a difficult task. The microphysical properties of nanometer‐scale nucleating clusters differ from those of bulk phases, a common phenomenon in nanoscience. For a long time it was believed that binary nucleation of H_2_SO_4_ and water vapor is the most important mechanism of new particle formation in the atmosphere, and today it is still considered to be important in the free troposphere. Although recent measurements show that ternary nucleation of H_2_SO_4_, water, and ammonia often dominates over binary nucleation [*Kirkby et al.*, 2011; *Glasoe et al.*, 2015; *Kürten et al.*, 2016], the understanding of the latter is crucial for regions of the free troposphere with low ammonia and for a deeper understanding of the fundamental processes involved in nucleation. Several approaches exist to describe binary nucleation, and in particular, classical nucleation theory has been established for a long time and is widely used. Improvements of the classical binary nucleation theory and a comparison with the CLOUD data are provided in *Duplissy et al.* [2016].

The approach used in the present study is based on detailed modeling of H_2_SO_4_ condensation and evaporation starting with the monomers [*Becker and Döring*, 1935]. This approach can be considered as an extension of the discrete general dynamics equation down to the monomer. Other examples of similar models include *McMurry and Friedlander* [1979], *Gelbard and Seinfeld* [1978], *McGraw and Marlow* [1983], *Yu* [2006], and *Kulmala* [2010]. We use the Sulphuric Acid Water Nucleation (SAWNUC) model of *Lovejoy et al.* [2004] which was not only originally developed for ion‐induced nucleation but also includes neutral nucleation. The evaporation rate of charged clusters containing a few H_2_SO_4_ molecules is based on measured thermodynamics [*Curtius et al.*, 2001; *Lovejoy and Curtius*, 2001; *Froyd and Lovejoy*, 2003]. For intermediate sizes the Gibbs energies are interpolated between these measured values and the bulk phase limit. Neutral nucleation is based on dimer and trimer thermodynamics [*Hanson and Lovejoy*, 2006; *Kazil and Lovejoy*, 2007] and bulk phase thermodynamics with adjustments to reproduce experimental results from *Ball et al.* [1999]. Three spin‐off models of SAWNUC exist that specifically aim to be used in climate modeling. The first one consists of a set of fitted equations by *Modgil et al.* [2005]. The second is the parameterized nucleation (PARNUC) model that uses parametrized condensation and evaporation rates and solves nucleation in steady state with a specialized solver [*Kazil and Lovejoy*, 2007], and based on that the third model uses a PARNUC look‐up table [*Kazil et al.*, 2010].

A direct comparison of experimental nucleation data from CLOUD with the independent results from the SAWNUC model is a tool to cross check and validate the model and the experimental measurements. Furthermore, the SAWNUC model can facilitate the understanding of the nucleation and growth processes. Here we compare the CLOUD binary nucleation measurements of the H_2_SO_4_‐H_2_O system at temperatures between 292 and 208 K with SAWNUC model results. The CLOUD project was established at CERN (European Organization for Nuclear Research) to improve the current understanding of nucleation, especially ion‐induced nucleation. The experiment consists of an aerosol chamber that can be illuminated by a particle beam from the CERN Proton Synchrotron (PS). The particle beam simulates galactic cosmic rays that are the major source of ions in the atmosphere. The chamber provides an extremely clean environment with state‐of‐the‐art technology. Contaminant levels of species that could contribute to the nucleation process are kept as low as possible, and the composition of charged nucleating clusters is continuously monitored so that the influence of such contaminants can be detected. Various instruments continuously record the chamber contents, such as precursor gas concentrations, particle concentrations, or molecular composition of charged clusters and operational parameters such as pressure, temperature, and rotational speed of the mixing fans.

Two sets of data are of special interest for the present study: the particle formation rates at a given diameter, here 1.7 nm (*J*_1.7_), and the distribution of the charged clusters. The charged cluster distribution is measured by an Atmospheric Pressure Interface Time of Flight (APi‐TOF) mass spectrometer [*Junninen et al.*, 2010]. This allows a direct comparison with the original work of *Curtius et al.* [2001], *Lovejoy and Curtius* [2001], *Froyd and Lovejoy* [2003], and *Lovejoy et al.* [2004]. Furthermore, in this study the peak formation rate of new particles, i.e., the maximum in the derivative of the total particle concentration *N* with respect to time (*∂*_*t*_ *N*), as measured by several condensation particle counters, CPCs, is compared with SAWNUC simulations. For this comparison the counting efficiencies of the instruments used at CLOUD are applied to the simulated aerosol and cluster populations. This allows a comparison with the measured CPC values without applying any corrections to calculate the formation rate at a smaller diameter (closer to the critical cluster size).

## 2 Methods

The original SAWNUC code [*Lovejoy et al.*, 2004] was modified to be applied to the CLOUD chamber experiments. Only a short summary of SAWNUC will be given here since it was described in detail by *Lovejoy et al.* [2004]. SAWNUC uses a mixture of experimentally determined Gibbs energies and interpolations to classical bulk phase thermodynamics to determine the sulphuric acid uptake and evaporation rates for each negatively charged and neutral cluster. Each cluster is assumed to be in equilibrium with the surrounding water vapor, an assumption that is valid for high water vapor concentrations found in the troposphere (see also *Schelling and Reiss* [1981] and *Jaecker‐Voirol et al.* [1987] for more details). SAWNUC determines the most probable water content of each cluster and defines the size of the cluster based on this water content. Figure S2 in the supporting information shows the water and sulphuric acid content as function of diameter. The actual sulphuric acid uptake and evaporation rate constants for a specific cluster with *i* H_2_SO_4_ is calculated as the weighted average over all water contents.

*Lovejoy et al*. [2004] fixed the ion‐ion recombination coefficient in the limit of small ions to be 1.6·10^−6^ cm^−3^ s^−3^. *Franchin et al.* [2015] indicate that the parameterization by *Brasseur and Chatel* [1983] is a better representation of ion‐ion recombination of small ions under low temperature and ground level pressure; therefore, this parametrization was used in this study. The system of differential equations describing the evolution of clusters and aerosols with *i* H_2_SO_4_ molecules is solved for either steady state conditions or time resolved. The steady state solution uses a direct iteration scheme that solves the set of differential equations, described in *Lovejoy et al.* [2004] and *Kazil and Lovejoy* [2007], for *∂*_*t*_ *n*_*i*_=0 and a maximum mobility equivalent diameter of 1.7 nm. Parameters used in SAWNUC are summarized in the supporting information Table S1.

In chamber experiments particles can be lost to the chamber walls. Mechanisms that transport particles to the wall are diffusion and gravitational settling. The latter can be ignored for nucleation experiments in the CLOUD chamber, since particles always remain smaller than 100 nm and gravitational settling is therefore negligible. *Ehrhart and Curtius* [2013] showed that the diffusion wall loss can have a significant influence on nucleation rates in chamber experiments. Rate coefficients for wall losses from each particle size bin *i* are taken into account by applying [*Crump et al.*, 1982; *Park et al.*, 2001; *Metzger et al.*, 2010]:

(1) $$k_{i}^{\text{wall}}=C_{\text{wall}}\;\sqrt{D_{i}}.$$

The size‐dependent diffusion coefficient *D*_*i*_ is assumed to be described by the Cunningham‐Millikan equation [*Cunningham*, 1910]. The coefficient *C*_wall_ includes turbulence due to flows induced by the mixing fans [*Voigtländer et al.*, 2012] and the geometry of the chamber. The value of *C*_wall_ at several temperatures was determined by wall loss experiments with H_2_SO_4_. Additionally, a constant first‐order dilution loss of (9.6·10^−5^ s^−1^) is taken into account due to the constant flow of gas through the chamber.

### 2.1 CLOUD Chamber

The CLOUD chamber is a stainless steel continuously stirred tank reactor, with a volume of 26 m^3^. A general description is given in the supplementary information of *Kirkby et al.* [2011] and in *Duplissy et al.* [2016]. The chamber is mixed by two fans that ensure short mixing times and an almost homogenous distribution of trace gases and nucleating particles [*Voigtländer et al.*, 2012]. Sulphuric acid is produced in situ by reaction of OH, SO_2_, and H_2_O vapor. The OH is generated in the chamber by photolysis of O_3_ and the subsequent reaction of O(^1^D) with H_2_O. O_3_ and SO_2_ mixing ratios vary between experiments in a range from a few ppbv to up to 3000 or 80 ppbv for O_3_ and SO_2_, respectively. The UV light for the photolysis is transmitted via a fiber optic system into the chamber to prevent additional heat; a detailed description can be found in *Kupc et al.* [2011]. The data used for our comparison were obtained during the CLOUD 3 (2010, originally published in *Kirkby et al.* [2011]) and CLOUD 5 (2011) campaigns. These campaigns were dedicated to binary and ternary (with NH_3_) nucleation. The main focus of CLOUD 5 was nucleation at low temperatures (208–248 K) characteristic of the upper troposphere.

### 2.2 Particle Dynamics and Counters

To simulate the dynamics of particle formation the set of equations describing the particle evolution is solved for time steps of typically 60 s with VODE (Variable Coefficient Ordinary Differential Equation solver) [*Brown et al.*, 1989]. To compare the model results directly with the observed temporal evolution of measured particle concentrations, the signals of various particle counters were modeled using their measured counting efficiencies [*Wimmer et al.*, 2013; *Riccobono et al.*, 2012] as a function of the mobility equivalent particle diameter $f\left(d_{p}^{m}\right)$ to the simulated aerosol population. These counting efficiencies are described either by a sigmoidal dependency on the particle diameter with four fit parameters, *p*_1_ to *p*_4_:

(2) $$f\left(d_{p}^{m}\right)=p_{1}+\frac{p_{2}}{1+\exp\left(\left(p_{3}-d_{p}^{m}\right)/p_{4})\right)}$$

or by the function from *Wehner et al.* [2011] with three parameters *a*, *b*, and *c*:

(3) $$f\left(d_{p}^{m}\right)=a-\exp\left(\left(b-\overset{m}{\underset{p}{d}}\right)/c\right).$$

The parameters used to model the particle counters are compiled in Tables 1 and 2. The particle diameter where 50% of particles are counted (*D*_50_) is also included in the table. The *D*_50_ is typically reported as a cutoff diameter. The counting efficiency is probably also a function of the temperature of the sampled aerosol. Although a first calibration at low temperatures has been performed to study the effect of sample temperature for some particle counters [*Wimmer et al.*, 2015], sufficient experimental data to account for this are currently not available.

**Table 1:** Parameters Used for the Counting Efficiency Described by Equation (3) From *Wehner et al.* [2011], Based on Calibrations With Different Chemical Species and Polarities [*Riccobono et al.*, 2012; *Wimmer et al.*, 2015]

CPC	Species	Charge	*a*	*b*	*c*	*D*_50_ (nm)
PSM 1	NaCl	‐	0.97	1.17	0.09	1.2
	NH_4_HSO_4_	‐	0.97	0.85	0.4	1.2
PSM 2	NaCl	‐	0.98	1.04	0.25	1.2
	NH_4_HSO_4_	‐	1.01	0.97	0.23	1.1
DEG 1	NaCl	‐	0.99	1.04	0.70	1.5
	NH_4_HSO_4_	‐	0.98	0.95	0.5	1.4
DEG 2	NaCl	‐	1.04	1.1	0.92	1.7
	NH_4_HSO_4_	‐	0.96	1.04	0.44	1.4
	H_2_SO_4_	‐	0.9	1.01	1.08	2.0
TSI 3776	WO_*x*_	‐	0.98	1.75	1.44	2.8
	WO_*x*_	+	1.0	1.97	1.74	3.2

**Table 2:** Parameters Used for the Counting Efficiencies Described by Equation (2), a

CPC	*p*_1_	*p*_2_	*p*_3_	*p*_4_	D_50_ (nm)
PSM 1	−0.003	0.95	1.7	0.06	1.6
PSM 2	0.015	0.96	1.70	0.07	1.6
DEG 1	−0.005	0.96	1.77	0.14	1.8

a All calibrations were performed with positively charged NH_4_HSO_4_ [*Wimmer et al.*, 2015].

Sampling line corrections, $l\left(d_{p}^{m},T\right)$, were applied to account for particle losses in the sampling tube [*Baron and Willeke*, 2001]. This correction is derived for straight sampling lines. Due to a lack of experimental characterization of the used Y splitter sampling lines, instruments connected with a Y splitter are corrected with the same method as for straight lines, although the losses are expected to be higher. For a CPC sampling through a Y splitter with a core sampling line, the individual losses for each part of the line are multiplied to yield an estimate of the total transmission efficiency,

(4) $$l_{t}\left(d_{p}^{m},T\right)=l_{y}\left(d_{p}^{m},T\right)\;\;l_{s}\left(d_{p}^{m},T\right)\;\;l_{c}\left(d_{p}^{m},T\right).$$

In case a Y splitter is used, the term *l*_*y*_ describes losses before splitting the flow and *l*_*s*_ describes losses after splitting the flow. For particle counters with straight sampling lines, *l*_*y*_=1, and *l*_*s*_ covers all losses in the sampling line up to the core sampling line *l*_*c*_.

The simulated particle number concentration, *N*_CPC_, that represents the uncorrected observed particle concentration rate with a CPC is

(5) $$N_{\text{CPC}}=\underset{i}{\sum }f\left(d_{p,i}^{m}\right)\;l_{t}\left(d_{p,i}^{m},T\right)\;n_{i},$$

where *n*_*i*_ is the number concentration for a cluster containing *i* H_2_SO_4_ molecules. The observed temporal evolution of the measured particle concentration is then given by

(6) $$\partial_{t}N_{\text{CPC}}=\underset{i}{\sum }f\left(d_{p,i}^{m}\right)\;l_{t}\left(d_{p,i}^{m},T\right)\;\partial_{t}n_{i}.$$

In order to minimize numerical errors, a linear increase in sulphuric acid content was applied, in steps of one molecule of H_2_SO_4_, for particles up to a mobility equivalent diameter of 3.3 nm. For particles larger than this, geometric size bins are applied. This ensures that all *D*_50_ step function counting efficiencies start to respond in the linear region of H_2_SO_4_ addition. For conversion from the mass diameter, *d*, that is used in SAWNUC to mobility equivalent diameter the relationship established by *Ku and Fernandez de la Mora* [2009], $d_{p}^{m}=d+0.3$ nm, was applied.

Relative humidity and temperature were kept constant during the individual nucleation events. The concentration of sulphuric acid was measured with a Chemical Ionization Mass Spectrometer (CIMS) [*Tanner et al.*, 1997; *Kürten et al.*, 2011, 2012] and time‐dependent H_2_SO_4_ concentrations are used as input for the SAWNUC modeling. Similarly, the time‐dependent ion pair production rate is used as an input parameter. The ion pair production rate is proportional to the number of ionizing particles that pass through the chamber each second [*Smirnov*, 2005; *Duplissy et al.*, 2010]. Ionizing particles come from galactic cosmic rays and the CERN PS pion beam. The pion (*π*) beam allows an adjustable ion pair production rate up to around 75 ion pairs cm^−3^ s^−1^. An electric field in the chamber with a potential difference of 60 kV can remove all ions efficiently in ≤ 1 s. Therefore, in all cases when the clearing field is switched on, *q*_ions_≈0.

#### 2.2.1 Determination of Peak Particle Formation Rate

During the CLOUD 5 campaign a battery of five particle counters with cutoff sizes, i.e., 50% probability of counting a particle with that size, ranging from 1.2 to 3 nm (mobility equivalent diameter) was operated. Four counters are diethylene glycol‐based CPCs, with two of them being continuous flow particle counters [*Iida et al.*, 2009; *Wimmer et al.*, 2013]. The other two are (mixing type) particle size magnifiers (PSM, model Airmodus A09) [*Vanhanen et al.*, 2011]. The fifth particle counter is a butanol‐based TSI 3776. Counting efficiencies as a function of particle diameter exist for all five particle counters. These counting efficiencies differ with chemical composition of the particles. Usual compounds used for calibrations are salts or metal oxides; therefore, calibrations with pure H_2_SO_4_‐H_2_O were not available, with the exception of a single calibration of the DEG‐2‐CPC. For details of these calibrations see *Wimmer et al.* [2013]. Recently, *Kangasluoma et al.* [2014] published a more detailed study on the influence of the chemical composition of aerosol particles on particle counter response functions. Additionally, the polarity of the aerosol particles has an influence on the counting efficiency [*Jiang et al.*, 2011; *Kuang et al.*, 2012] and *Chen* [2013] showed that neutral particles have counting efficiencies different from charged particles. The measured *∂*_*t*_ *N* is compared with the simulated one by comparing the maximum values and the time *t*_max_ at which this maximum occurs. During nucleation experiments in the CLOUD chamber the temporal evolution of the particle concentration, *∂*_*t*_ *N*(*t*), will reach a maximum before dropping to zero when formation of new particles and losses of particles reach a steady state. The region around this maximum is approximated with a Taylor series truncated after the second‐order term. The method for peak formation rates works best for single nucleation experiments as it was the standard procedure during CLOUD 5 rather than consecutive experiments, i.e., an experiment where nucleation conditions are changed from neutral to Galacatic Cosmic Rays (GCR) and then to pion beam without a cleaning cycle in between [cf. *Kirkby et al.*, 2011, Figure S1].

## 3 Results

### 3.1 Comparison With CLOUD Particle Formation Rates

For the binary experiments during CLOUD 3, a data set of particle formation rates at 1.7 nm (mobility equivalent diameter) exists [*Kirkby et al.*, 2011]. For CLOUD 5 these data are summarized in *Kürten et al.* [2016]. These data sets contain steady state particle formation rates that were corrected for losses [*Kürten et al.*, 2015, 2016].

**Figure 1:**
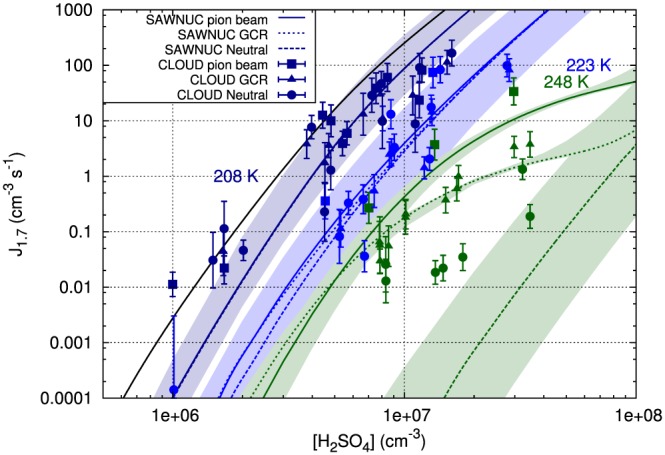
Experimentally determined (symbols) and modeled (lines and shaded area) formation rates of 1.7 nm particles versus H_2_SO_4_ at temperatures below 273 K. The lines represent SAWNUC modeling for conditons with the *π* beam on (solid line), GCR (dotted), and neutral (dashed line) conditions. The area shown for the SAWNUC modeling represents variability due to a shift of 0.5 kcal mol^−1^ in the Gibbs energies. Squares represent experiments with ion production by the beam, while triangles are under GCR conditions; circles represent neutral experiments. Different colors show different temperatures which are given in the plot next to the shaded areas. The black line shows a potential enhancement in the condensation rate constants due to van der Waals interaction described by a Hamaker potential.

**Figure 2:**
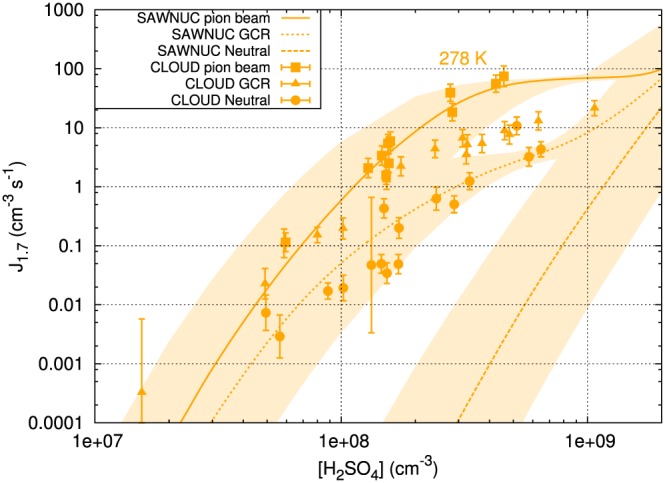
As Figure 1 but for 278 K. Differences between modeled and measured particle formation rates at neutral and GCR conditions are assumed to be mainly due to NH_3_ contamination.

**Figure 3:**
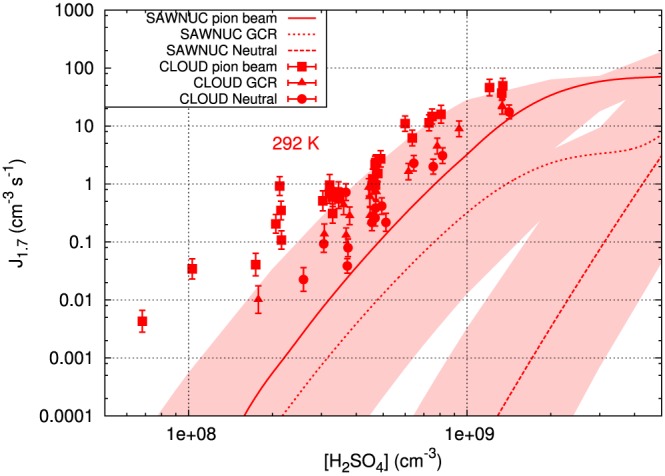
As Figure 1 but for 292 K. Differences between modeled and measured particle formation rates at neutral and GCR conditions are assumed to be mainly due to NH_3_ contamination.

An overview of the measured and modeled particle formation rate as function of H_2_SO_4_ when no NH_3_ is added is shown in Figure 1 for temperatures below 273 K, in Figure 2 for 278 K, and in Figure 3 for 292 K. For the calculated results the shaded area shows the variability due to a variation in the Gibbs energies, which were varied by doing two additional calculations, in one case adding and in the second case subtracting 0.5 kcal mol^−1^ to the cluster Gibbs energies of stepwise sulphuric acid addition. This approach is similar to the variation done by *Lovejoy et al.* [2004]; however, *Lovejoy et al.* [2004] used random normally distributed errors of the Gibbs energies, while we used an absolute shift for each step. The choice of an absolute shift was made here to see the maximum change that could occur. *Lovejoy et al.* [2004] introduced this as a reasonable ad hoc assumption to explore the changes that are caused by such an assumption. As the absolute uncertainties are not known we also use a shift of 0.5 kcal mol^−1^.

**Figure 4:**
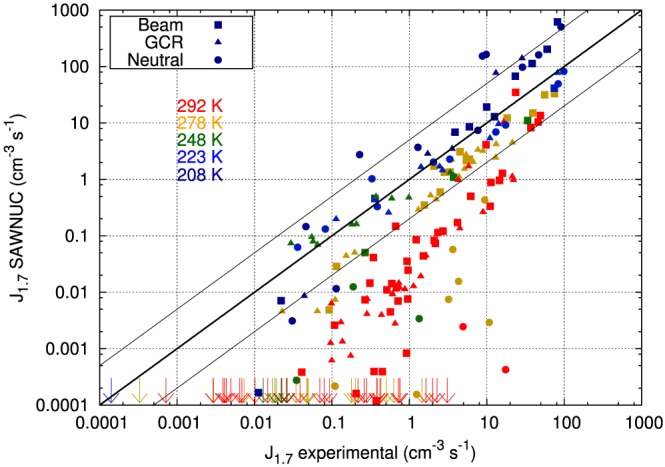
Correlation between *J*_1.7_ simulated with SAWNUC and *J*_1.7_ based on measurements for all temperatures and relative humidities. At temperatures of 292 and partly also at 278 K the experimental results are biased high due to the influence of ammonia contamination on the observed nucleation rates. The solid black line corresponds to a 1:1 agreement; the thinner lines parallel to it indicate deviation by a factor of 5 and 0.2.

The model results are all derived for a relative humidity of 38% and the ion pair production rate is assumed to be 75 ion pairs cm^−3^ s^−1^ at 278 K in the case of pion beam nucleation (solid lines in Figures 1, 2, 3) and 4 ion pairs cm^−3^ s^−1^ at 278 K for GCR nucleation (dotted lines). It is important that GCR in this context does not mean ionization only by galactic cosmic rays but also ionization by residual radiation from the CERN PS that penetrates the chamber and thus increases the ion pair production rate. The ion pair production rates were scaled by the density of the chamber air at different temperatures.

Although NH_3_ was not added during these experiments at 278 K and 292 K the ammonia contamination in the CLOUD chamber was considerable (the ammonia background concentration was around 2 pptv and 4 pptv) and the nucleation process was strongly influenced by ammonia. This is confirmed by the APi‐TOF measurements that show the presence of ammonia in the nucleating clusters see *Kürten et al.* [2016], *Duplissy et al.* [2016], and *Schobesberger et al.* [2015] for a comprehensive discussion of the NH_3_ background in the CLOUD chamber. The contaminant ammonia most likely explains the difference in nucleation rates for the experimental (ternary) data and the binary model results at the warmer temperatures. At 248 K still some NH_3_ was detected during the experiments; however, the background was much lower (0.3 pptv) and less ammonia was present in the APi‐TOF data. It should be noted that for 208 K and 223 K the relative humidity in the CLOUD chamber differed from 38%, but at these temperatures the influence of relative humidity is relatively low and does not affect the overview character of Figure 1. Figure S1 in the supporting information gives an indication of the humidity dependence for the five temperatures shown here. The black line in Figure 1 shows the possible enhancement of the particle formation rates due to attractive interactions between neutral particles. The attractive potential was described by the Hamaker potential [*Hamaker*, 1937] assuming a Hamaker constant of 6.4·10^−13^ erg [*Chan and Mozurkewich*, 2001]. SAWNUC originally does not include any enhancements in the monomer uptake and coagulation rates of neutral species. Since the parametrization of the neutral channel to the results of *Ball et al.* [1999] was done without this, a reparametrization would be necessary but such a reparametrization is outside the scope of this paper.

At 292 K, Figure 3, the deviation between model and experiment is more pronounced than at 278 K. The simulated results at 278 K in Figure 2 show that at $\left[H_{2}{\text{SO}}_{4}\right]=1\cdot 10^{9}$ cm^−3^ the neutral nucleation exceeds the ion‐induced nucleation, which reached its plateau at the value of the ion pair production rate. At lower temperatures the agreement between SAWNUC and experimental results is better as the influence of ternary nucleation decreases. The decreasing influence can mainly be attributed to a significantly lower background concentration of NH_3_ at these temperatures. At temperatures below 248 K no ammonia was detected in the nucleating clusters, indicating that the ammonia had condensed to the walls [*Kürten et al*., 2016]. However, the dependence of the particle formation rate to the Gibbs energies decreases with decreasing temperature, due to the exponential dependency of the evaporation rate

(7) $$k_{i}^{d}=k_{i-1}^{a}\;\rho^{0}\exp\left(\beta \Delta_{R}G_{i}^{0}\right).$$

Here $\Delta_{R}G_{i}^{0}$ is the standard reaction Gibbs energy for the formation of cluster *i* from cluster *i* − 1, $k_{i-1}^{a}$ is the association rate constant for the cluster containing one H_2_SO_4_ less, *ρ* ^0^ is the standard density, and $\beta =\frac{1}{k_{b}T}$ is the reciprocal temperature. This exponential temperature dependence of the evaporation rate constant dominates over the weak $\sqrt{T}$ dependence of the forward rate constant and therefore controls the temperature dependence of the nucleation rate.

Figure 1 shows that at low temperatures the particle formation rates can be higher for lower ion pair production rates. As already shown in *Kazil and Lovejoy* [2004], this is mainly because of the reduced lifetime due to higher recombination rates of ions at high total ion concentration. At low total ion concentration losses to the wall are the dominant loss term for ions.

Figure 4 gives a direct comparison between experimental and simulated formation rates. For the correlation plot the same conditions as in the experiments were used in the simulation. It can be seen that overall there is good agreement between particle formation rates from SAWNUC and particle formation rates from CLOUD for temperatures between 208 to 278 K in the presence of ions (i.e., GCR and with the pion beam). For temperatures of 208 and 223 K neutral nucleation agrees well with the model, while the deviations at 248, 273, and 292 K are most likely due to ammonia contamination.

Arrows in Figure 4 indicate data points that are outside of the given axis range. This is the case for neutral nucleation at temperatures of 278 and 292 K (as mentioned in the discussion of Figures 2 and 3). A comparison for all the data is included in Table 3 that contains the parameters for linear least squares fits to $\log\left(J_{\text{sim}}\right)$ versus $\log\left(J_{\exp}\right)$ for the various temperatures and neutral as well as ion‐induced nucleation. In case of perfect agreement the slope would be 1 and the intercept 0. Therefore, these fits give a direct indication how good the agreement between experimental and simulated result is.

**Table 3:** Linear Fits to the Data in Correlation Plot in Figure 4, a

*T* in K	Type	Size	Slope	Intercept	*R* ^2^
208.15	n	8	1.3 ± 0.2	0.32 ± 0.26	0.86
208.15	ch	11	1.5 ± 0.07	−0.15 ± 0.1	0.98
208.15	n and ch	19	1.4 ± 0.09	0.07 ± 0.13	0.93
222.15	n	11	1.1 ± 0.14	−0.16 ± 0.19	0.86
222.15	ch	10	0.87 ± 0.06	−0.043 ± 0.06	0.97
222.15	n and ch	21	1 ± 0.08	−0.12 ± 0.1	0.88
248.15	n	7	1.7 ± 0.61	−1.8 ± 0.78	0.54
248.15	ch	14	0.7 ± 0.05	−0.24 ± 0.04	0.94
248.15	n and ch	21	0.73 ± 0.08	−0.25 ± 0.06	0.82
278.15	n	23	2.1 ± 0.28	−3.2 ± 0.25	0.71
278.15	ch	33	1.1 ± 0.054	−0.58 ± 0.06	0.93
278.15	n and ch	56	1.3 ± 0.1	−0.78 ± 0.11	0.76
292.15	n	16	2.3 ± 0.19	−6.6 ± 0.16	0.9
292.15	ch	46	2.2 ± 0.2	−2.4 ± 0.22	0.73
292.15	n and ch	62	2.2 ± 0.18	−2.5 ± 0.2	0.71
208‐222	n and ch	50	1.3 ± 0.07	0.00 ± 0.09	0.89

a In the column labeled *Type*, *ch* indicates a run with the clearing field off (i.e., ions present), while *n* indicates results with the clearing field on (no ions present). The column *size* gives the size of the data set used in the fit.

### 3.2 Comparison With Peak Particle Formation Rates

**Figure 5:**
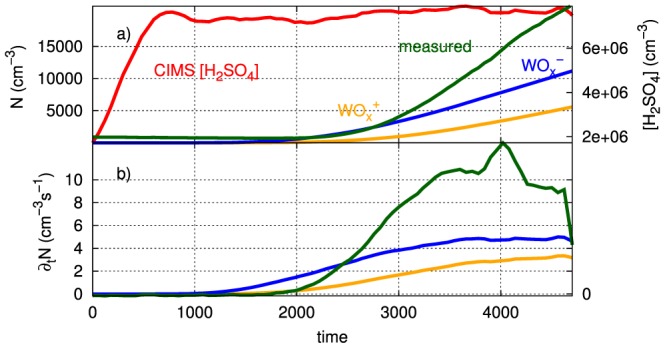
(a) Observed particle concentration during a typical CLOUD experiment as measured by a CPC 3776 (green line) and compared to simulation with SAWNUC for the expected count rate of a CPC with the counting efficiency curve determined with negatively and positively charged tungsten oxide (blue and orange, respectively) and H_2_SO_4_ concentration (red); (b) measured and simulated particle formation rates, *∂*_*t*_ *N* for the same CPC as in Figure 5a.

**Figure 6:**
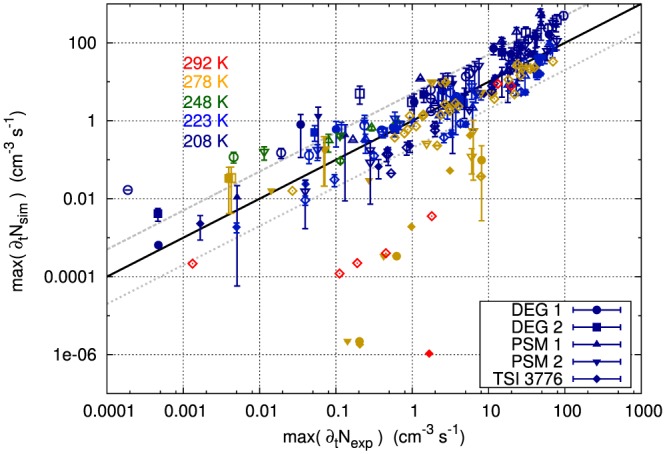
Comparison of simulated and measured peak formation rates for different particle counters. Data points for $\max(\partial_{t}N_{\text{sim}})$ are averaged over all counting efficiencies for a given CPC. Open symbols indicate experiments with ions in the chamber; solid symbols indicate neutral experiments. The error bars represent upper and lower values caused by different counting efficiencies curves for the same particle counter. The black line indicates a one‐to‐one agreement between simulation and observation; the grey lines indicate deviation by a factor of 5 and 0.2.

Figure 5 shows that the number concentration and *∂*_*t*_ *N* can differ between two counting efficiencies. Due to this difference, the average of the maximum *∂*_*t*_ *N* over all counting efficiencies is used to compare simulation and experiment for the CLOUD 5 data set. To take into account the different counting efficiencies, the upper and lower limits of the peak formation rates are used as uncertainties of the simulated particle formation rate. In general, simulated and measured particle formation rates agree well for temperatures below 273 K (see Figure 6). For most of the particle counters the agreement between experimental and simulated values is within a factor of 5 for temperatures at or below 278 K.

At temperatures of 278 K and above, NH_3_ contamination has a major influence on nucleation, especially for neutral nucleation. Here SAWNUC predicts peak particle formation rates which are considerably lower than the measured ones. Furthermore, the simulations did, in cases with relatively large cutoff diameters, not reach a maximum formation rate before the end of the simulated experiment. This is especially a problem in case of low nucleation and growth rate conditions and particularly with NH_3_ contamination (i.e., the experiment resulted in a quick nucleation, while a binary system would have not responded within that time). Therefore, the direct comparison with the measured time series of H_2_SO_4_ and ion pair production rate is limited to “well behaved” runs, i.e., runs with an additional waiting period after the particle formation rate peaked. At higher temperature NH_3_ contamination is higher and has a major influence on nucleation, especially for neutral nucleation. Here SAWNUC predicts particle formation rates which are considerably lower than the measured ones.

### 3.3 Comparison of the Charged Cluster Distribution

**Figure 7:**
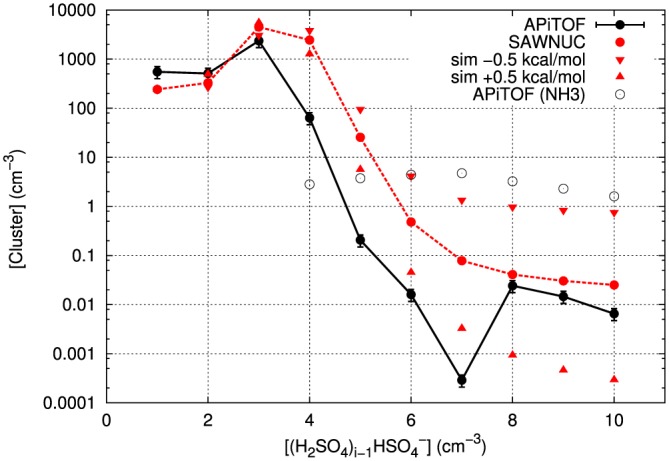
Steady state cluster distribution as measured by the APi‐TOF (black) and simulated with SAWNUC (red circles) (top) at 292 K, [H_2_SO_4_] = 3.3·10^8^ cm^−3^ s^−1^ and RH = 38% as a function of the number of sulphur atoms in the cluster. The red triangles show the result of an ±0.5 kcal mol^−1^ shift in the Gibbs energies of the clusters. The open black circles give the total number concentration of clusters containing at least one ammonia molecule.

**Figure 8:**
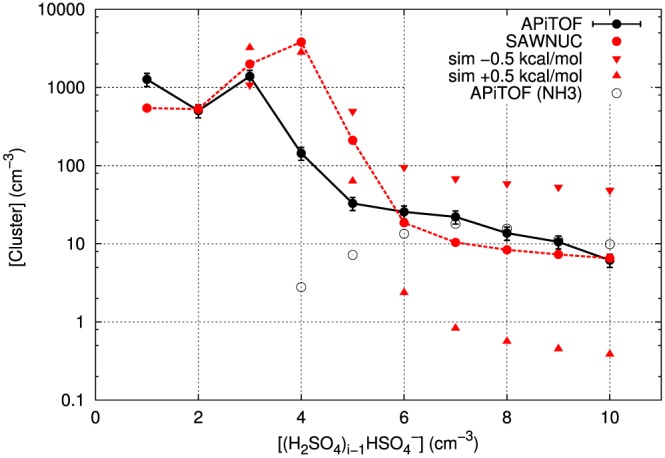
Steady state cluster distribution as measured by the APi‐TOF and simulated with SAWNUC (top) at 278 K, [H_2_SO_4_] = 1.6·10^8^ cm^−3^ s^−1^ and RH = 36%. The red triangles show the result of an ±0.5 kcal mol^−1^ shift in the Gibbs energies of the clusters. The open circles give the total number concentration of clusters containing at least one ammonia molecule.

**Figure 9:**
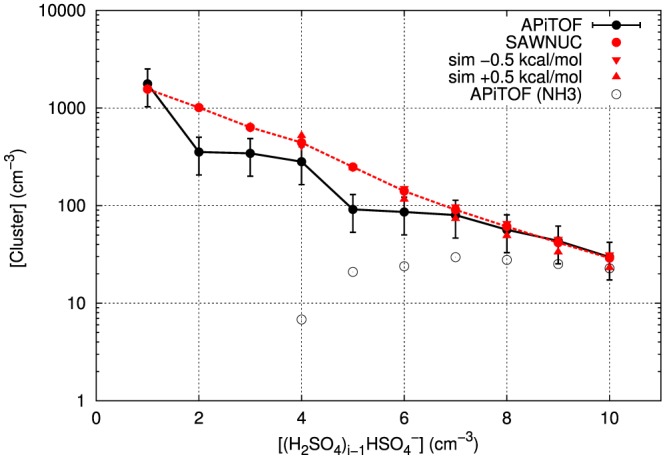
Steady state cluster distribution as measured by the APi‐TOF and simulated with SAWNUC (top) at 248 K, [H_2_SO_4_] = 3·10^7^ cm^−3^ s^−1^ and RH = 33%. The red triangles show the result of an ±0.5 kcal mol^−1^ shift in the Gibbs energies of the clusters. The open circles give the total number concentration of clusters containing at least one ammonia molecule.

The APi‐TOF provides an important insight into the distribution of charged clusters in the CLOUD chamber. A comparison of measured charged cluster distributions at different temperatures with a simulated distribution is shown in Figures 7, 8, 9. The comparison includes only concentrations from CLOUD 3 at temperatures from 292 K to 248 K as at lower temperatures for conditions in CLOUD 5 a reliable conversion from count rates to absolute concentrations is not available. For high temperatures (292 K, Figure 7) the position of the measured and simulated maxima both appear at the cluster containing three sulphuric acid molecules. The modeled clusters include a certain water content, for each cluster this water does not appear in the measured clusters due to evaporation inside the APi‐TOF (see below). At 278 K (Figure 8) the measured peak position is decreased by one H_2_SO_4_ molecule compared to the SAWNUC result. This can be explained by fragmentation of the clusters inside the APi‐TOF (as discussed below) or errors in the experimental Gibbs energies used in SAWNUC. *Lovejoy et al.* [2004] used an uncertainty in the Gibbs energies of ± 0.5 kcal mol^−1^. To show the sensitivity of the distribution, this value is used as upper and lower bounds in the calculation. Variations in the Gibbs energy within the applied ±0.5 kcal mol^−1^ could partially explain the shift in the maximum of the cluster distribution, but not the difference of almost an order of magnitude between the simulated and modeled ${\left(H_{2}{\text{SO}}_{4}\right)}_{3}\cdot {\text{HSO}}_{4}^{-}$. Also the high ${\text{HSO}}_{4}^{-}$ concentrations indicate that the most likely cause of the discrepancy is fragmentation inside the APi‐TOF.

**Figure 10:**
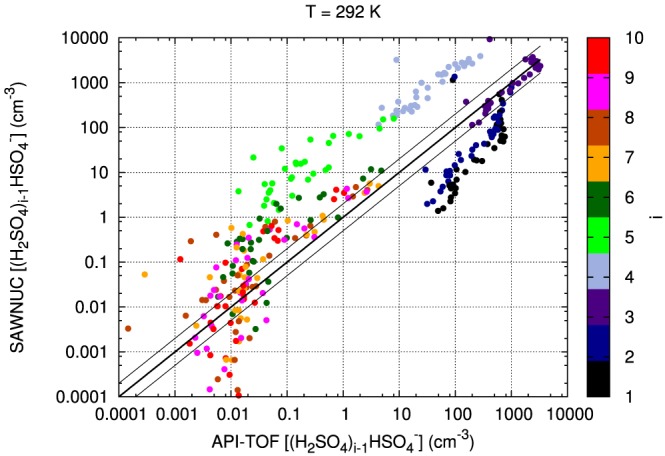
Correlation plot showing calculated cluster concentration versus measured cluster concentration at 292 K. The composition of each cluster is color coded by the number *i* of sulphuric acid molecules in the clusters ${\left(H_{2}{\text{SO}}_{4}\right)}_{i-1}\cdot {\text{HSO}}_{4}^{-}$. The decreased concentration for larger cluster and increased concentration of smaller cluster in the APi‐TOF are indications for fragmentation. Error bars were omitted for sake of clarity.

**Figure 11:**
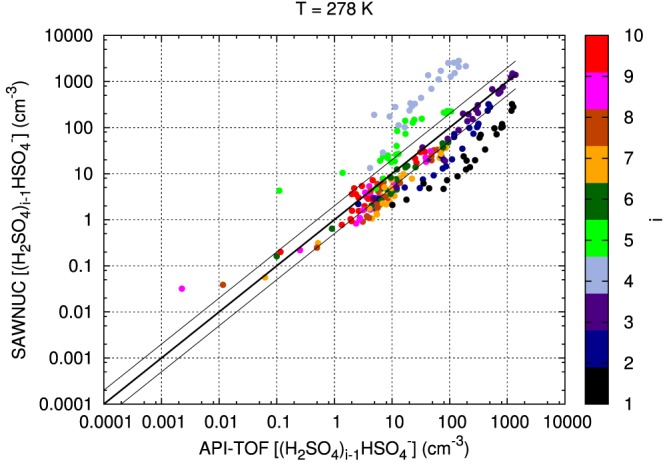
Correlation plot showing calculated cluster concentration versus measured cluster concentration at 278 K. The composition of each cluster is color coded by the number *i* of sulphuric acid molecules in the clusters, ${\left(H_{2}{\text{SO}}_{4}\right)}_{i-1}\cdot {\text{HSO}}_{4}^{-}$. The fragmentation pattern is similar as in Figure 10 with the difference that for larger clusters SAWNUC and the APi‐TOF measurements seem to agree better.

**Figure 12:**
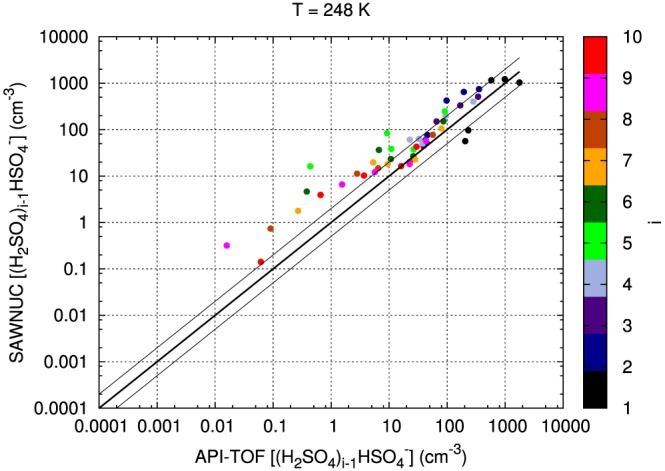
Correlation plot showing calculated cluster concentration versus measured cluster concentration at 248 K. The composition of each cluster is color coded by the number *i* of sulphuric acid molecules in the clusters ${\left(H_{2}{\text{SO}}_{4}\right)}_{i-1}\cdot {\text{HSO}}_{4}^{-}$. No clear fragmentation pattern is visible here.

Figures 10, 11, 12 show calculated steady state cluster concentrations versus the cluster concentration measured with the APi‐TOF. The number of sulphuric acid molecules in each cluster is color coded. These figures show the data for the whole CLOUD 3 campaign, at temperatures from 292 K to 248 K. From Figures 10 and 11 it is evident that SAWNUC predicts lower concentrations of the smallest clusters and higher concentrations of clusters containing between four and eight sulphuric acid molecules (with the ${\text{HSO}}_{4}^{-}$ core ion included in that number). A possible explanation is evaporation and fragmentation in the mass spectrometer. When entering the low pressure region of the first stage of the APi‐TOF, the pressure drops to 0.002 bar. After an initial adiabatic cooling, the clusters will be heated up again by collisions with residual gas molecules in the first stage and acceleration by electric fields in the atmospheric pressure interface. This process will lead to a substantial energy deposition into the clusters. A larger cluster should be able to dissipate this energy into more vibrational modes of freedom. Furthermore, larger clusters contain more water molecules when entering the mass spectrometer. The initial evaporation of these water molecules can cool down the cluster. The high ${\text{HSO}}_{4}^{-}$ concentration is the result of fragmentation of clusters containing a few H_2_SO_4_ molecules. Fragmentation does not occur by stepwise evaporation of H_2_SO_4_ thus enabling ${\left(H_{2}{\text{SO}}_{4}\right)}_{i-1}\cdot {\text{HSO}}_{4}^{-}$, with *i*≥4, to fragment to ${\text{HSO}}_{4}^{-}$ and $(H_{2}{\text{SO}}_{4})\cdot {\text{HSO}}_{4}^{-}$ without having the relatively stable ${\left(H_{2}{\text{SO}}_{4}\right)}_{2}\cdot {\text{HSO}}_{4}^{-}$ as intermediate. The dependence of possible fragmentation reactions is further complicated by a possible dependence on the initial state of the charged clusters. For example, at 248 K there is no clear fragmentation pattern visible. A possible explanation is that the clusters entering the mass spectrometer do not heat up fast enough, from the relatively low temperature of their initial state, to allow for fragmentation. Furthermore, relatively stable clusters do probably not fragment in the APi‐TOF, for example, the $(H_{2}{\text{SO}}_{4})\cdot {\text{HSO}}_{4}^{-}$ appears to be stable enough to be detected and is the largest fragmentation product. The same is probably true for very stable clusters containing dimethyl amine, such as the clusters described in *Kürten et al.* [2014].

Evaporation of clusters can also occur inside the sampling line during the transition from the chamber to the mass spectrometer. The depletion of H_2_SO_4_ in the sampling line, due to losses to the walls, results in a lower sulphuric acid uptake of the clusters and shifts the expected equilibrium cluster distribution. It is therefore possible that clusters evaporate toward a new distribution during the sampling process. Whether the clusters reach the new steady state distribution depends on their evaporation rate and the dwell time in the sampling line. However, such an evaporation cannot explain the high concentrations of HSO ${}_{4}^{-}$ and $(H_{2}{\text{SO}}_{4})\cdot {\text{HSO}}_{4}^{-}$ seen in Figures 10 and 11.

The presence of ammonia can also lower the concentration of measured clusters larger than ${\left(H_{2}{\text{SO}}_{4}\right)}_{3}\cdot {\text{HSO}}_{4}^{-}$ compared to the SAWNUC model. In this case the clusters containing several sulphuric acid and the bisulphate ion will take up ammonia and would therefore appear in a different channel in the APi‐TOF. However, it is difficult to explain the very high ${\text{HSO}}_{4}^{-}$ signal in the APi‐TOF if only NH_3_ uptake would be responsible for it. This does not exclude the possibility that ammonia containing clusters fragment in the APi‐TOF and leave ${\text{HSO}}_{4}^{-}$ as the detected fragment. Additionally, the influence of ammonia for the charged cluster distribution is already much smaller at 278 K, and also, there is a ${\text{HSO}}_{4}^{-}$ signal present in the APi‐TOF that is clearly elevated compared to the abundance expected from SAWNUC.

Besides fragmentation, another reason for the observed discrepancies in the cluster distribution could be an underestimation of the uncertainties in the mass‐dependent transmission efficiencies of the APi‐TOF mass spectrometer. If the transmission efficiencies varied strongly over the mass range between the ${\text{HSO}}_{4}^{-}$ and $(H_{2}{\text{SO}}_{4})\cdot {\text{HSO}}_{4}^{-}$ signals, leading to an overestimation of the HSO ${}_{4}^{-}$ signal relative to the signals from the larger clusters, then the observed cluster distribution could result. Further technical studies should be made on transmission functions of the APi‐TOF mass spectrometer [*Junninen et al.*, 2010; *Heinritzi et al.*, 2015].

## 4 Summary and Conclusions

The direct comparison of binary H_2_SO_4_‐H_2_O aerosol formation between the CLOUD measurements and the SAWNUC model over the temperature range of 208 to 292 K yields several conclusions. First, the derived nucleation rates agree well for the temperature range of 208–223 K, for ion‐induced and neutral nucleation. Nucleation rates at temperatures below 230 K show only a small dependence on the evaporation rates. However, they still exhibit some dependence on evaporation rates and provide an important limit for the Gibbs energies of the neutral and charged clusters. At 248 and 278 K the modeled neutral nucleation rates are substantially smaller than the measured ones, but the ion‐induced nucleation rates are described well by SAWNUC, especially for high ion pair production rates (pion beam). This demonstrates that the evaporation rates of the ion clusters are described adequately in SAWNUC and implicitly confirms also the experimental results of *Curtius et al.* [2001], *Lovejoy and Curtius* [2001], and *Froyd and Lovejoy* [2003] that provide the thermochemical data for the ion‐induced nucleation path in SAWNUC. The GCR nucleation rates at 278 K still agree reasonably well but the CLOUD rates are typically a factor 5 higher than SAWNUC predictions most likely due to contaminant ammonia concentrations of a few pptv. Here the nucleation mechanism transitions into a ternary nucleation.

At the warmer temperatures of 278 K and more pronounced at 292 K, the experimental nucleation rates are found to be substantially higher than the predictions from SAWNUC. At 292 K the experimental rates are at least a factor 10 higher than expected by SAWNUC for binary nucleation and often several orders of magnitude higher. Here the measured nucleation is a ternary nucleation where SAWNUC can only provide lower limits of the nucleation rate.

Considering the enormous range (many orders of magnitude) of binary nucleation rates that had been reported for comparable conditions in the literature [cf. *Benson et al.*, 2008, and references therein], the agreement between SAWNUC and CLOUD over a range of temperatures and H_2_SO_4_ concentrations is very good (i.e., for temperatures of 208–223 K for neutral nucleation and for 208–278 K for ion‐induced nucleation). Note that the SAWNUC model is entirely independent of the CLOUD measurements and there are no free parameters in the model.

Second, we conclude that SAWNUC can be used to model directly the particle concentration measurements of a CPC during nucleation. The comparison works reasonably well but is limited especially by the uncertainties of the detection efficiency function of the CPC as a function of particle size, chemical composition, and charging state.

Third, the sulphuric acid ion cluster distributions modeled by SAWNUC compared reasonably well with APi‐TOF measurements at 248 K. As an important difference at temperatures of 278 and 292 K, the monomer concentration was generally found to be unexpectedly high in the APi‐TOF. This is most likely explained by the fragmentation or evaporation of larger clusters in the APi‐TOF or by inaccuracies of the mass‐dependent transfer function.

## 5 Outlook

In a future study, SAWNUC should be extended to take into account ternary vapors. The results of *Froyd and Lovejoy* [2012] should be included together with the development of SAWNUC to include ternary or general multicomponent nucleation. Before such studies can be conducted a detailed sensitivity study must be performed to define the most important parameters for determining the free energies. The optimization of such parameters should be done by fitting them to tabulated ternary particle formation rates from clean experiments, e.g., *Zollner et al.* [2012], *Glasoe et al.* [2015], and CLOUD data. Although the present CLOUD data already cover a large range of temperatures and H_2_SO_4_ concentrations, additional measurements that are free of NH_3_ are needed to allow a detailed direct comparison of SAWNUC and CLOUD at warm temperatures (∼292 K). The humidity dependence of binary neutral and ion‐induced nucleation also needs to be studied in more detail in future CLOUD experiments.

## Supporting information



Supporting Information S1

Supporting Information S2

Figure S1

Figure S2
