# Supplementary material for: Comparison of the SAWNUC model with CLOUD measurements of sulphuric acid‐water nucleation
Source: J Geophys Res Atmos. 2016 Oct 27;121(20):12401–14. doi: 10.1002/2015JD023723 (PMC5302017; doi:10.1002/2015JD023723)
Supplement: Supplementary file 2 — Supporting Information S2 [file JGRD-121-12,401-s002.pdf]

Supporting Information for "Comparison of the SAWNUC model with CLOUD measurements of sulphuric acid-water nucleation"

Sebastian Ehrhart,[1,2] Luisa Ickes,[1,*] Joao Almeida,[1,2] Antonio Amorim,[3] Peter Barmet,[4] Federico Bianchi,[4,*] Josef Dommen,[4] Eimear M. Dunne,[5,6] Jonathan Duplissy,[7,8] Alessandro Franchin,[7] Juha Kangasluoma,[7] Jasper Kirkby,[1,2] Andreas Kürten,[1] Agnieszka Kupc,[9] Katrianne Lehtipalo,[4,7] Tuomo Nieminen,[7,8] Francesco Riccobono,[4] Linda Rondo,[2] Siegfried Schobesberger,[7,+] Gerhard Steiner,[7,9,10] António Tomé,[3] Daniela Wimmer,[1,7] Urs Baltensperger,[4] Paul E. Wagner,[9] Joachim Curtius[1]

1

Goethe-University Frankfurt am Main, Institute for Atmospheric and Environmental Sciences, Altenhoeferallee 1, 60438 Frankfurt am Main, Germany

2

CERN, 1211 Geneva, Switzerland

3

SIM, University of Lisbon and University of Beira Interior, 1749-016 Lisbon, Portugal

4

Paul Scherrer Institute, 5232 Villigen, Switzerland

5

School of Earth and Environment, University of Leeds, LS2 9JT, UK

6

Finnish Meteorological Institute, Atmospheric Research Centre of Eastern Finland, PL 1627, 70211 Kuopio, Finland

[7]

Department of Physics, P.O. Box 64, FI-00014 University of Helsinki, Finland

[8]

Helsinki Institute of Physics, P.O.Box 64, FI-00014 University of Helsinki, Finland

[9]

Faculty of Physics, University of Vienna, Boltzmanngasse 5, 1090 Vienna, Austria

[10]

Institute of Ion Physics and Applied Physics, Leopold-Franzens University, Innsbruck Technikerstraße 25, 6020 Innsbruck, Austria

[*]

Now at: Institute for Atmospheric and Climate Science, ETH Zurich, Switzerland

[+]

Now at: Department of Atmospheric Sciences, University of Washington, Seattle, WA 98195, USA

Contents of this file

1. Figures S1 to S4

Text S1. The SAWNUC model was originally described in *Lovejoy et al.* [2004]. Table S4 summarises the current state of SAWNUC and provides the literature sources containing further details. The reaction schemes, forward reaction rate constants and evaporation rate constants presented there are still used, with the following exceptions:

- The evaporation rates of neutral particles was adjusted in *Kazil and Lovejoy* [2007].
- Instead of calculating a diurnal cycle of H_2SO_4 the measured sulphuric acid concentrations are used.
- The ion-ion recombination rate constant for small ions from *Brasseur and Chatel* [1983] was used.
- A wall loss term was added as first order loss term

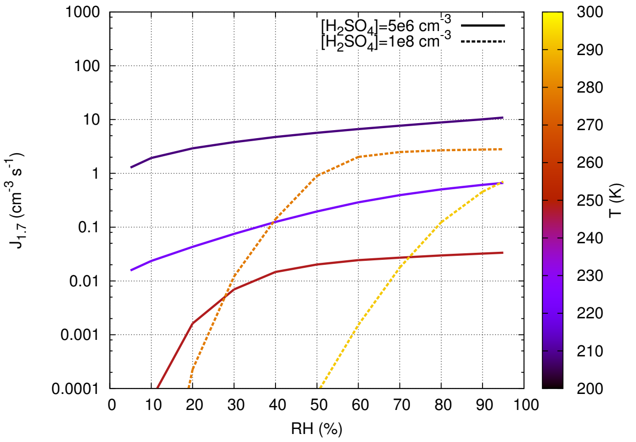

Figure S2. Modelled particle formation rate as function of relative humidity under GCR conditions in CLOUD (4 ion pairs cm^{-3} s^{-1}). The solid line gives results for temperatures below 273 K while the dashed lines give results for warmer temperatures. The temperature is also given in the colour scale. For the colder temperatures the sulfuric acid concentration was $5 \cdot 10^6$ cm^3 and for the warmer temperatures $1 \cdot 10^8$ cm^{-3}.

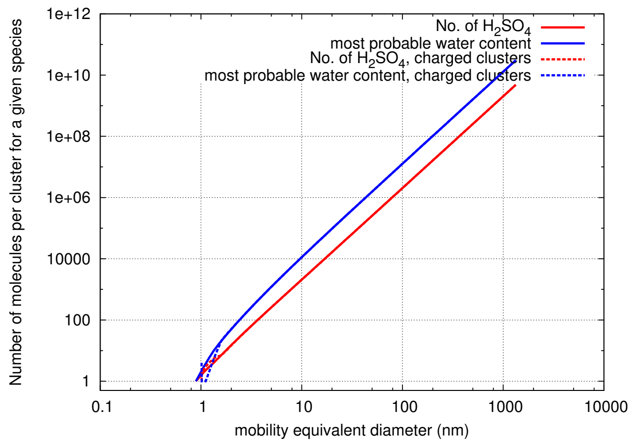

Figure S3. Number of sulphuric acid and water molecules in the particles as function of diameter for neutral particles (solid lines) and charged particles (solid lines) at 278 K and 40% relative humidity.

Table S4. Summary of SAWNUC:

Max. cluster diameter for steady state calculation	1.7 nm
Max. diameter linear size bin	3.3 nm
Geometric scaling factor	1.15
Diameter of a particle	fixed H_2SO_4 and most probable water content
Most recent description of :	
neutral thermodynamics	*Kazil and Lovejoy* [2007]
charged thermodynamics	*Lovejoy et al.* [2004]
H_2SO_4 uptake	*Lovejoy et al.* [2004]; *Kazil and Lovejoy* [2007]
recombination	*Brasseur and Chatel* [1983]; *Franchin et al.* [2015]
Ions :	
Negative	$(H_2SO_4)_i{\cdot}HSO_4^-$
Positive	$(H_2O)_5{\cdot}H_3O^+$

References

Brasseur, G., and A. Chatel (1983), Modeling of stratospheric ions: a first attempt,, *Annales Geophysicae*, *1*, 173–185, doi:doi:10.1016/0032-0633(82)90101-5.

Franchin, A., S. Ehrhart, J. Leppä, T. Nieminen, S. Gagné, S. Schobesberger, D. Wimmer, J. Duplissy, F. Riccobono, E. M. Dunne, L. Rondo, A. Downard, F. Bianchi, A. Kupc, G. Tsagkogeorgas, K. Lehtipalo, H. E. Manninen, J. Almeida, A. Amorim, P. E. Wagner, A. Hansel, J. Kirkby, A. Kürten, N. M. Donahue, V. Makhmutov, S. Mathot, A. Metzger, T. Petäjä, R. Schnitzhofer, M. Sipilä, Y. Stozhkov, A. Tomé, V.-M. Kerminen, K. Carslaw, J. Curtius, U. Baltensperger, and M. Kulmala (2015), Experimental investigation of ionion recombination under atmospheric conditions, *Atmospheric Chemistry and Physics*, *15*(13), 7203–7216, doi:10.5194/acp-15-7203-2015.

Kazil, J., and E. R. Lovejoy (2007), A semi-analytical method for calculating rates of new sulfate aerosol formation from the gas phase, *Atmospheric Chemistry and Physics*, *7*(13), 3447–3459, doi:10.5194/acp-7-3447-2007.

Lovejoy, E. R., J. Curtius, and K. D. Froyd (2004), Atmospheric ion-induced nucleation of sulfuric acid and water, *J. Geophys. Res. - Atmospheres*, *109*, D08,204.
