# Supplementary figures and images for: Comparison of the SAWNUC model with CLOUD measurements of sulphuric acid‐water nucleation
Source: J Geophys Res Atmos. 2016 Oct 27;121(20):12401–14. doi: 10.1002/2015JD023723 (PMC5302017; doi:10.1002/2015JD023723)

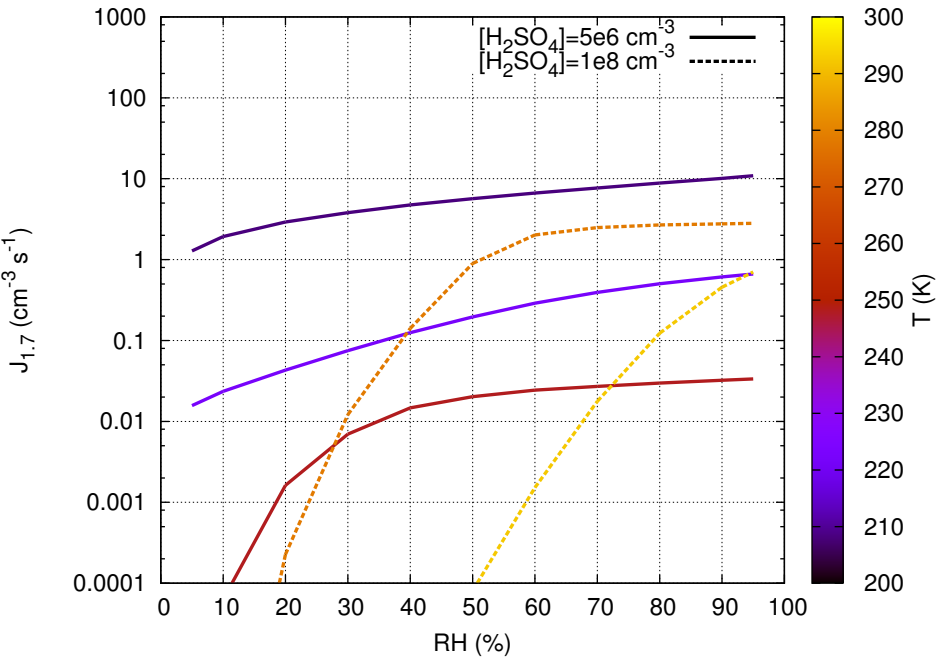

Supplement: Supplementary file 3 — Figure S1 [file JGRD-121-12,401-s003.pdf]

Number of molecules per cluster for a given species

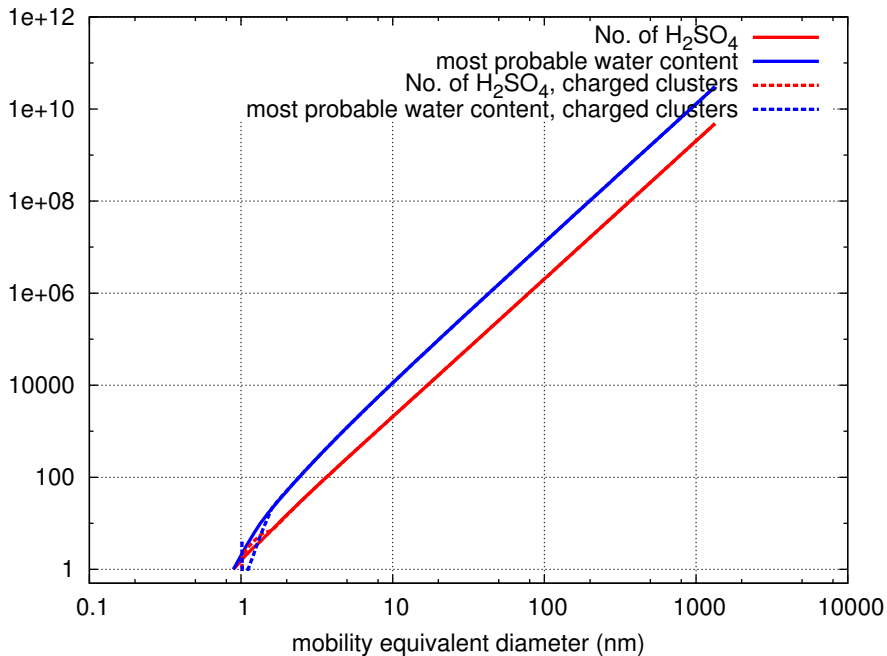

Supplement: Supplementary file 4 — Figure S2 [file JGRD-121-12,401-s004.pdf]
